# Ophthalmic Miscellany

**Published:** 1874-08-15

**Authors:** A. D. Williams

**Affiliations:** St. Louis, Mo.


### OPHTHALMIC MISCELLANY.

By A. D. Williams, M.D., St. Louis, Mo.

UNDER this head I desire to mention, very briefly, a few cases of interest I have recently seen. I refer, first, to a couple of cases of

### MUSCULAR ASTHENOPIA.

Asthenopia literally means a weakness of vision; but it does not imply a defect in the acuteness or power of vision, but an inability on the part of the individual to use his eyes continuously. They fatigue or tire, and actually give out after reading or looking at small objects for a short time.

In muscular asthenopia the weak vision results from a loss of balance in the external muscles of the eyes. A glance at a case or two will enable us to understand the nature of the trouble better than a mere description.

Case 1.—Mr. M., a young man, cannot use his eyes sufficiently for the wants of a medical student. Some years since he had to quit school because he could not use his eyes, and has not been able to use them since.

He could see perfectly for a few moments, but his eyes would soon tire and trouble him so much that he had to give up all literary pursuits.

His chief complaint to me was, that as soon as his eyes would begin to tire, the print would all run together and mix up so that he could not see any of it distinctly. By examination I found that the acuteness of vision was normal, and that the refraction was normal; that is, he was neither far-sighted nor near-sighted.

By having the patient fix some small distant object, and covering first one eye and then the other, and observing the covered eye, it was discovered that each eye would deviate slightly outwards under the cover. This proved that there was slight tendency to external strabismus, resulting from defective action or insufficiency of probably both internal recti muscles. When he would converge the eyes for a few moments, as he must do in accommodating as in reading, the internal straight muscles, being too weak, would soon give way and the eyes would diverge slightly in spite of the will. This divergence would cause the print to partially double, which would make it seem to run together and mix up so as to make the whole very indistinct.

In this way the chief symptom in his complaint was produced. I prescribed prismatic glasses No. 3, and had the bases placed directly inwards, so as to favor both internal recti muscles; that is to say, the effect of the prisms enabled him to accommodate without having to converge the eyes as much as without them. Thus these muscles are favored by the action of the prisms.

The young man put on his prismatic glasses, and, notwithstanding the constant use of his eyes in studying, he has had no trouble since. Had not the glasses relieved him, it would have become necessary to perform tenotomy on one or both of the external muscles.

Case 2.—Mr. V., middle-aged man, has always been short-sighted, but No. 10 concave glasses make his vision perfect for distance. Has been wearing glasses, but says he cannot wear them constantly because they make his head ache.

Having the patient fix a small object in the distance, and covering the eyes in succession, I found that each eye deviated slightly inwards under the cover. This showed that there was a slight tendency to convergent strabismus, on account of insufficiency of the external recti muscles, exactly the opposite condition shown in the above case. Upon examining the glasses he had been wearing, I found that they were placed too far apart, so that the visual line from each eye passed through the inner edge of each glass. This position of the glasses produced a prismatic effect, but, unfortunately, this favored the internal muscles, which were already too strong. This accidental position of the glasses, of course, only increased his trouble. In looking in the distance without the glasses, and more so with them, as he had them, the external recti muscles were not sufficient to hold the eyes straight, consequently they were constantly converging very slightly. This produced a constant tendency to double vision. This caused an unnatural nervous impression upon the brain, which resulted in the constant headache mentioned above.

I told him to have his glasses set close together, so that each eye would look through the external margin of the glasses, in order that the prismatic effect of the glasses thus arranged would favor the external recti muscles, which are defective or insufficient. The effect of arranging the glasses in this way is the same as if prisms were placed before the eyes with their bases outwards. At the same time the concave glasses correct the myopia.

The patient followed directions, and has worn his glasses ever since without the least trouble of any kind. Had not the glasses thus arranged relieved the trouble, tenotomy of one or both of internal recti would have been necessary.

It will be observed that the internal recti in the first case, and the external recti in second case, were at fault. The first patient could not read because the internal muscles were not sufficient to converge the eyes continuously. The second had trouble in looking in the distance, because the external muscles were not sufficient to hold the eyes parallel continuously. This was particularly the case when the glasses were so placed as to produce a prismatic effect in favor of the already too powerful internal muscles, and consequently increased the deficiency of the external muscles to the same extent. Muscular asthenopia is an interesting subject, but I will go no further into details here.

